# Study on Compatibility and Rheological Properties of High-Viscosity Modified Asphalt Prepared from Low-Grade Asphalt

**DOI:** 10.3390/ma12223776

**Published:** 2019-11-17

**Authors:** Mingliang Li, Feng Zeng, Ruigang Xu, Dongwei Cao, Jun Li

**Affiliations:** 1 Road Engineering Research Center, Research Institute of Highway Ministry of Transport, Xitucheng Road, No. 8, Haidian District, Beijing 100088, China; jun.li@rioh.cn; 2 China Road & Bridge Corportation, Andingmenwai Street, No. 88, Beijing 100011, China; zengf@crbc.com; 3 China Railway Third Bureau Group Co., Ltd. Survey and Design Branch, Jinsong Road, No. 16, Yingze District, Taiyuan 030001, Shanxi Province, China; 4 Zhonglugaoke (Beijing) Road Technology Co. Ltd., Xitucheng Road, No. 8, Beijing 100088, China; caodongwei@vip.126.com

**Keywords:** high-viscosity modified asphalt, low grade asphalt, porous asphalt, rheological property, viscosity-temperature characteristic

## Abstract

High-viscosity modified asphalt is mainly used as a binder for porous asphalt in China and Japan. In order to meet the demand for using porous asphalt under high temperature condition in Africa, high-viscosity asphalt made from low-grade matrix asphalt, which is commonly used in Africa is investigated. Based on simulation of local climate in Africa, the suitable range of high viscosity additive content for different matrix asphalt was obtained by analyzing dynamic viscosity of the asphalt. Through PG high temperature grading, multi-stress repeated creep, accelerated fatigue, temperature sweep and other tests, changes of high temperature, anti-fatigue and anti-shear indicators before and after modification were compared and analyzed and effects of different matrix asphalt were also studied. Finally, considering engineering requirements, mixing and compaction temperatures of various high-viscosity modified asphalt were determined through study of viscosity-temperature characteristics. This research provides a support for preparation of high-viscosity modified asphalt and porous asphalt mixture by using low grade asphalt. The research achievements can help to guide the material design and application of porous asphalt in Africa and other high temperature areas.

## 1. Introduction

Compared with dense-graded asphalt pavement, porous asphalt has the advantages of reducing water mist in rainy days, increasing driving safety, anti-skid, noise reduction and effectively alleviating urban heat island effect because of its large voids content. However, during the application of porous asphalt, performance of the pavement is highly dependent on materials and environment where it is used. When studying the performances of porous asphalt, it is necessary to considering the combined effect of traffic, the climate of the project site, as well as the material properties. 

At the beginning of the application of porous asphalt, straight run asphalt or natural asphalt was mainly used as binders. For example, 40/60 straight-run was generally used in the United States. Due to the low viscosity of such binders, porous asphalt was easily damaged, which limited its promotion. And many countries began to use modified asphalt, which greatly improved service life of porous asphalt [1]. Based on improvement and optimization of European experience and according to climate and traffic characteristics of various regions in the country, the Japanese road engineering community focused on study of binders and proposed high-viscosity modified asphalt (HVMA). And it became a key technology for high temperature stability and anti-raveling of porous asphalt in the country [2,3,4]. At present, HVMA is also use as binder for porous asphalt in China and the dynamic viscosity at 60 °C can be over 100,000 Pa·s [5]. High-viscosity modified asphalt can be prefabricated by blending matrix asphalt and high-viscosity additive (HVA), which is called “wet method” of modification and the technology by putting HVA directly during mixing process of mixture is called “dry method.” The HVA is generally a particle shape, with a length between 2~5 mm. It can be melted and dispersed rapidly and evenly in the mixing process of asphalt mixture.

In previous studied, it has been revealed that the main component of HVA is thermoplastic rubber, which forms a polymer network structure between the polymer in the HVA and the asphalt component [6], which generally results a higher viscosity in comparison of SBS modified asphalt. Qin et al. used temperature sweep and frequency sweep tests to investigate the influence of temperature and frequency on anti-rutting performance of high-viscosity modified asphalt and the methods are considered better presents a practical load in the road surface [7]. Tan et al. studied the composite modification of matrix asphalt using a thermoplastic elastomer and SBS polymer modified asphalt. It found that the thermoplastic elastomer particles as high elastic interlocking units are uniformly distributed in the network structure in the modified asphalt, which leads to good high and low temperature performance based on Performance Grade (PG) grading [8]. Cai et al. carried out research on environmental friendly alternative binders for permeable asphalt mixture by recycling engineering wastes including crumb rubber powder and recycled oil to prepare high-viscosity asphalt binders. The results showed that the performance can meet the specification requirements in China [9]. Xu et al. investigated the ageing mechanism of the high-viscosity asphalt and developed rejuvenator material for preventive maintenance [10]. In addition, studies were also performed on the noise reduction properties of porous asphalt using HVMA as a binder [11,12]. However, it showed that the high-viscosity binder mainly improves the mechanical performance of the pavement and has less influence of sound absorption. In existing studies, the high-viscosity asphalt are mainly made from matrix asphalt with grade 70 or composite modification of SBS asphalt. The compatibility of low-grade asphalt, the penetration at 25 °C of which is generally 30~50 (0.1 mm), for preparing high-viscosity asphalt are not studied yet. And there is also little knowledge on performances of HVMA made from low grade asphalt.

This paper focuses on rainy environments in Africa, which is characterized by high temperature in summer and heavy rainfall in spring and summer. When used in such conditions, theporous asphalt mixture should have sufficient resistance to high temperature stability, moisture damage and structural durability. As the average lowest temperature in such areas are higher than 20 °C, the low temperature properties are not taken into account in this study. At the same time, different from existing studies in China and Japan, matrix asphalt used in Africa is mainly low-grade asphalt due to high temperatures in summer. Besides, the performance and chemical composition of matrix asphalt from different sources are also quite different. Therefore, study is carried out for verifying the feasibility of preparing high-viscosity modified asphalt based on low-grade matrix asphalt and the performances of these high-viscosity asphalt used for African highways are investigated. The research will provide a technical support for using HVMA in Africa and other areas with high temperatures in summer and it contributes to promotion and application of porous asphalt based on HVMA.

## 2. Materials and Methods Study on Optimum Mixing Content of HVA

The mixing proportion of HVA is the main factor affecting the performance of high-viscosity modified asphalt. Therefore, the optimum mixing content of HVA for four low-grade matrix asphalt commonly used in Africa was studied first. 

### 2.1. Matrix Asphalt

The indicators of the four types of matrix asphalt used in West Africa are shown in Table 1. The four types of asphalt are numbered B1, B2, B3 and B4 respectively. Tests were carried out according to European Standard BS EN12591-2009. The chemical analysis results of the four-component tests are shown in Table 2. It can be seen from Table 1 and Table 2 that asphaltene content in B1 and B4 asphalt is high, resulting in relatively high softening point but penetration index after aging is relatively low. Asphaltene content in B3 asphalt is the lowest, so its softening point is relatively low but performance declines least after aging.

### 2.2. Preparation of HVMA

(1) Test equipment

**Figure 1:**
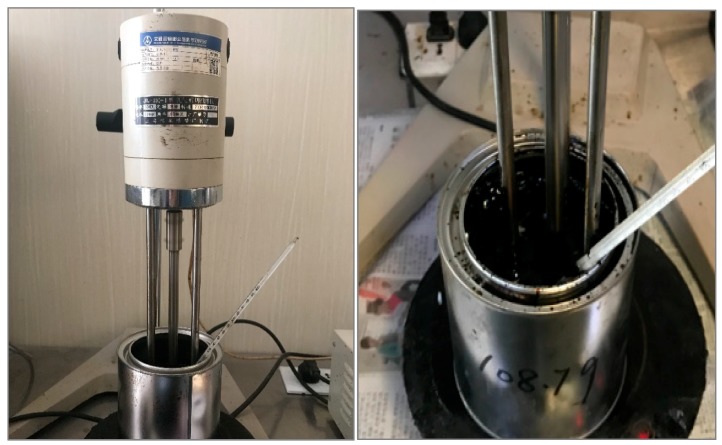
Preparation of HVMA using high-speed shear mixer.

The equipment required for preparation of high-viscosity asphalt binder includes high-speed shear mixer, heating furnace, thermometer, glass stirring rod. Preparation of high-viscosity asphalt using high-speed shear mixer is shown in Figure 1.

(2) Preparation Process

Preparation of high-viscosity modified asphalt in laboratory is as follows:

(1) Take a certain amount of matrix asphalt and heat the asphalt to about 180 °C or 190 °C (for SBS modified asphalt), then weigh the asphalt. Calculate the amount of high-viscosity additive according to the proportion of the designed content and add it into the asphalt, mix it evenly with glass rod;
(2) Place the sample cup under the high-speed shear machine, set rotation speed to 5000 rpm and shearing shall be continued for 30 min. The temperature during the whole process is controlled between 180 °C and 190 °C;
(3) After shearing and blending, place the prepared HVMA in an oven at 180 °C for 30 min. Then take it out for various tests.

### 2.3. Selection of Key Indicators for High-Viscosity Asphalt Binder

Existing studies show that [5], with the increase of dynamic viscosity of asphalt at 60 °C, compressive strength, splitting strength and bending strength of porous asphalt mixture are obviously increased. Besides, dynamic stability and other road performance are also significantly improved. A reasonable value of dynamic viscosity at 60 °C ensures porous asphalt to be used under high temperature conditions and without big increase in cost. Therefore, it is necessary to analyze the combined effect of temperature, load and dynamic viscosity on rutting performance of porous asphalt.

Among them, temperature and load are external factors, while dynamic viscosity is an internal factor and dominates the effect. According to existing research [5], the relation between dynamic stability of high-viscosity modified asphalt mixture and dynamic viscosity is as follows:

(1) $$\mathrm{lgDNS}=0.535\mathrm{lg}\eta -0.0653t-0.63p+5.592\left(R^{2}=0.892\right),$$

where,

DNS—Rutting test dynamic stability, times/mm;
*η*—Asphalt dynamic viscosity at 60 °C, Pa·s;
*t*—Test temperature, °C;
*p*—Loading pressure, MPa.

**Figure 2:**
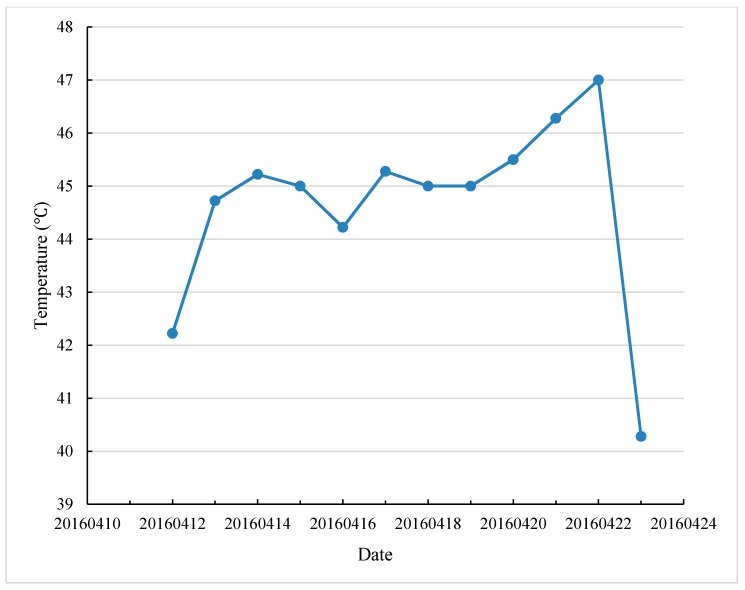
Average maximum temperature for 7 consecutive days over 30 years in certain African region (Data are from the reference [13]).

At present, 60 °C is usually chosen as test temperature to evaluate high temperature performance of asphalt mixture in many countries. According to meteorological data of some countries in Africa, the project is located in low latitude area and its climate is humid, hot and rainy, with high temperature in summer. The average maximum temperature for 7 consecutive days can reach 45.5 °C, as shown in Figure 2.

Calculation formula for design temperature of pavement is given in SHRP research [14], as shown by Equation (2). The temperature used to calculate PG high temperature grade is that of 2 cm below road surface.

(2) $$T_{20mm}=T_{air}-0.00618Lat^{2}+0.2289Lat+42.2\times 0.9545-17.78,$$

where:

T_20 mm_—the maximum temperature at 2 cm below road surface (°C);
*T_air_* —average maximum air temperature for 7 consecutive days (°C);
*Lat*—local latitude (°).

According to Equation (2), the highest design temperature of local pavement is 67.9 °C. Therefore, evaluation of high temperature performance of asphalt and mixture at 60 °C does not reflect actual working condition of pavement in summer. In this paper, considering a long life design of the pavement, 70 °C is used as the temperature for evaluating ultimate temperature performance of mixture and 0.7 MPa is used as load for rutting test load. In order to meet the needs of pavement material design for application, the dynamic stability of porous asphalt is not less than 3000–5000 times/mm. Considering an application for light traffic case, this paper suggests that 3000 times/mm is quite reasonable. Calculated by Equation (1), the required dynamic viscosity of asphalt at 60 °C shall be no less than 260,124 Pa·s. Then 270,000 Pa·s is chosen as lower limit of dynamic viscosity at 60 °C, so as to ensure the performance of the mixture at locally high temperatures.

At the same time, as HVMA has greater viscosity than ordinary modified asphalt, construction workability must be considered in evaluating performance of modified asphalt. A large number of engineering practices show that Brookfield viscosity of high-viscosity modified asphalt at 170 °C shall not exceed 3 Pa·s [5].

### 2.4. Influence of HVA Content on Performance of Asphalt

**Figure 3:**
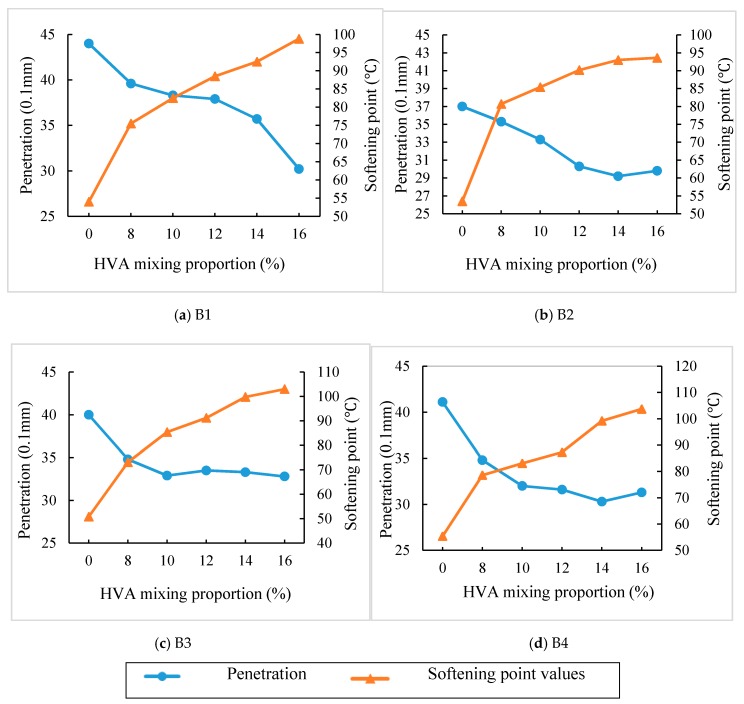
Penetration, softening point values of four HVMA with different HVA mixing proportion.

**Figure 4:**
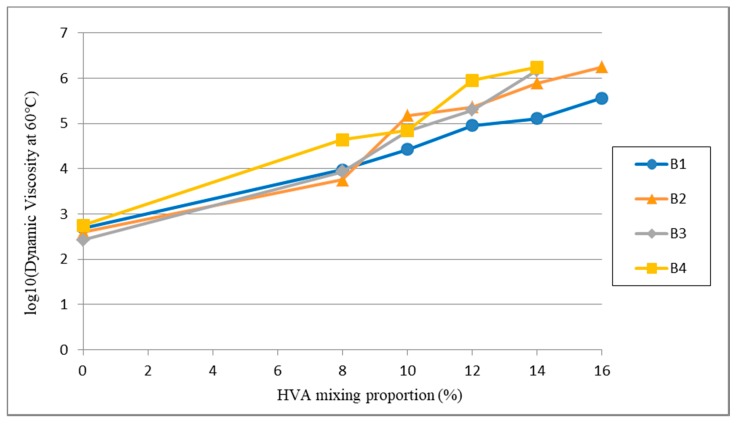
Dynamic viscosity at 60 °C of asphalt with different HVA mixing proportions.

Firstly, high-viscosity asphalt samples are prepared in the lab by using the four kinds of different base materials (as shown in Table 1 and Table 2) and HVA with different contents. Then performances of high-viscosity asphalt are tested, including penetration, softening point, dynamic viscosity at 170 °C and dynamic viscosity at 60 °C. Variations of these parameters caused by different HVA contents are analyzed as well. Five different mixing contents of HVA are used, namely 8%, 10%, 12%, 14% and 16%. The relationship between penetration and softening point of high-viscosity asphalt prepared by different matrix asphalt and HVA content is shown in Figure 3. Dynamic viscosity at 60 °C for different HVA content is shown in Figure 4. For better illustration of the tendency of the dynamic viscosity changing with the HVA content, the dynamic viscosity at 60 °C is shown in logarithmic form.

(1) Penetration

In many countries, penetration is regarded as an index for asphalt grade, which reflects the asphalt consistency. It can be seen from Figure 3 that the penetration of the four matrix asphalt after modification is significantly reduced, which is mainly due to absorption of light components in asphalt by HVA addition. It makes the asphalt thicken and become harder. When HVA content increases from 0% to 14%, penetration values for B1 to B4 matrix asphalt reduces by 19%, 21%, 17% and 26%, respectively. In terms of reduction in penetration, B4 is more sensitive to HVA modification.

(2) Softening point

As shown in Figure 3, the softening point of the four modified asphalt increases with the HVA content but the increasing trends slow down after it rises to 14%. Compared with matrix asphalt, the softening points of the four asphalt are all above 90 °C when 14% HVA is added.

(3) Dynamic viscosity at 60 °C

In Figure 4, it shows that dynamic viscosities of the four modified asphalt increase exponentially with HVA content. For asphalt B4, when HVA content is 8%, its dynamic viscosity at 60 °C reaches 43,914 Pa·s and its dynamic viscosity is the most sensitive to mixing content of HVA. The variation on dynamic viscosity at 60 °C of asphalt B1 is less affected by content of HVA, which shows a poor compatibility between the matrix asphalt and the modifier at current contents.

### 2.5. Determination on Optimum Range of HVA Content 

**Figure 5:**
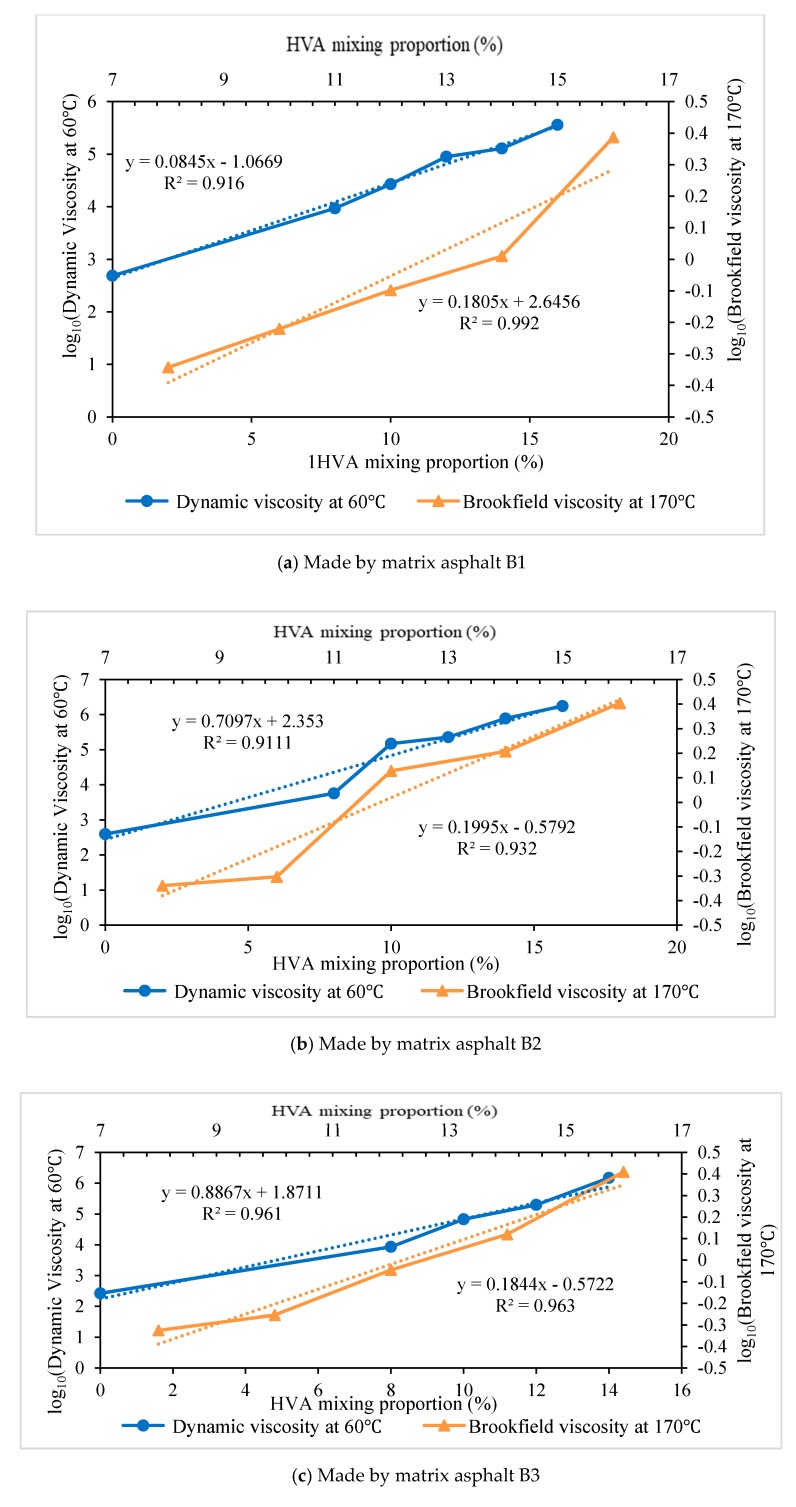

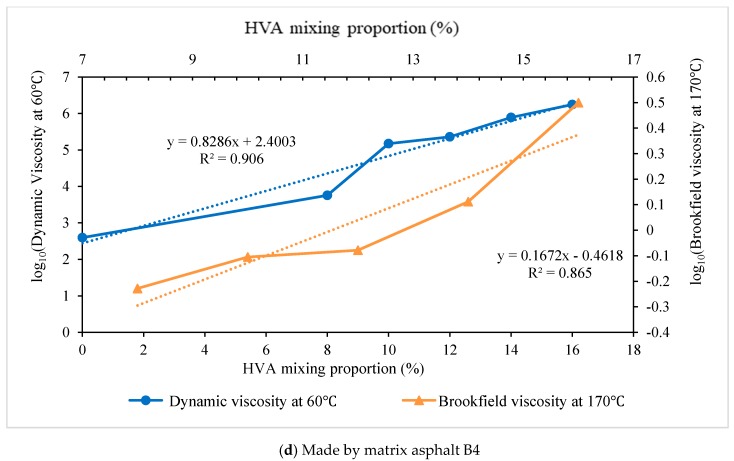
Dynamic viscosity and Brookfield viscosity for different HVA mixing proportion.

Based on test results, dynamic viscosity at 60 °C and Brookfield viscosity at 170 °C of different HVMA are correlated with HVA mixing proportion and regression curves are shown for expressing the relationships in Figure 5. From Figure 5, it can be seen that when expressed in logarithmic form, there are good linear relationships between the dynamic viscosity at 60 °C and HVA contents, as well as for Brookfield viscosity at 170 °C. 

According to analysis in Section 2.3, Brookfield viscosity at 170 °C (not more than 3.0 Pa·s) is used as an index to control maximum content of HVA and dynamic viscosity at 60 °C (not less than 270,000 Pa·s) is used as index to determine minimum content of HVA. The proper ranges of HVA content corresponding to different matrix asphalt are calculated based on the regression relationship in Figure 5. The results for B1–B4 are respectively: 15.5%~18.2%, 12.5%~16.6%, 12.3%~17.3% and 11.0%~17.2%.

## 3. Experimental Study on Rheological Properties of High-Viscosity Modified Asphalt 

In this section, rheological properties of HVMA made from various matrix asphalt are studied. In order to facilitate the investigation and considering the cost effective for using HVA in practical engineering, the HVA content 14% is used for the high-viscosity modified asphalt preparation, namely the ratio between the mass of asphalt and HVA is 86:14. For matrix asphalt B1, dynamic viscosity at 60 °C after mixing of 14% HVA cannot meet the requirement in Section 2.3, so it is not further studied in the experimental research on rheological properties in this paper. 

### 3.1. High Temperature Performance Grade

High temperature performance grade (PG) is calculated from complex shear modulus |G*| and phase angle δ, which are measured by dynamic shear rheometer. In this section, dynamic shear rheometer (DSR) is used for the test. The experimental parameters are set to be 10 rad/s as angular frequency, 1 mm above and below of parallel plate, 12% of strain value for original asphalt and 10% for aged asphalt. In the Strategic Highway Research Program (SHRP) research program, rutting resistance is characterized by rutting factor |G*|/sinδ. PG grading requires |G*|/sinδof original asphalt to be greater than or equal to 1.0 kPa and |G*|/sinδof short-term aging residue to be greater than or equal to 2.2 kPa. PG grading of asphalt sample is carried out and the test results are shown in Table 3. 

It can be seen from Table 3 that: The PG grade for matrix asphalt B2, B3 and B4 are 70, 64 and 70 respectively. After modified by 14% HVA, there are increases by three grades for the three types of matrix asphalt, which means great improvement of high temperature performance. PG high temperature grade of B3 + 14% HVA can reach PG82 which is the highest temperature grade and that of the other two even exceeds the highest grade.

### 3.2. Asphalt Temperature Sweep Test 

**Figure 6:**
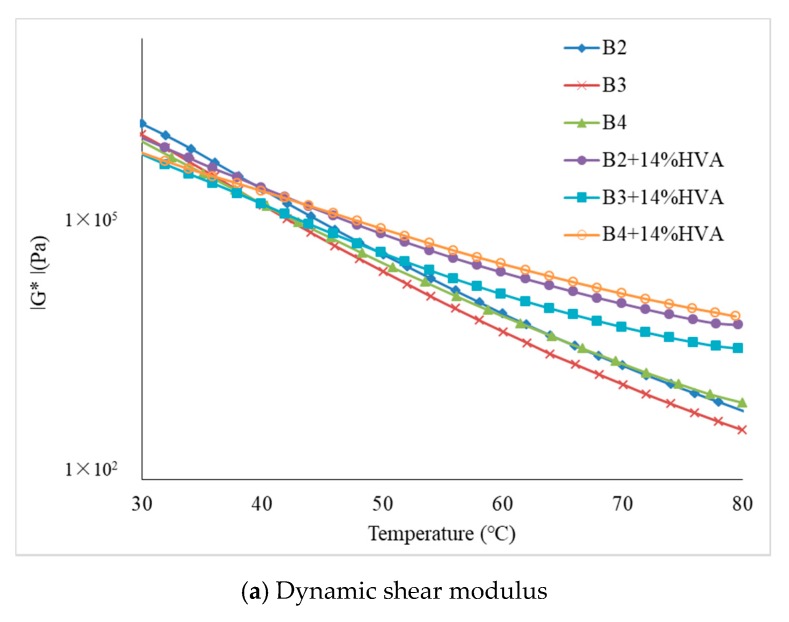

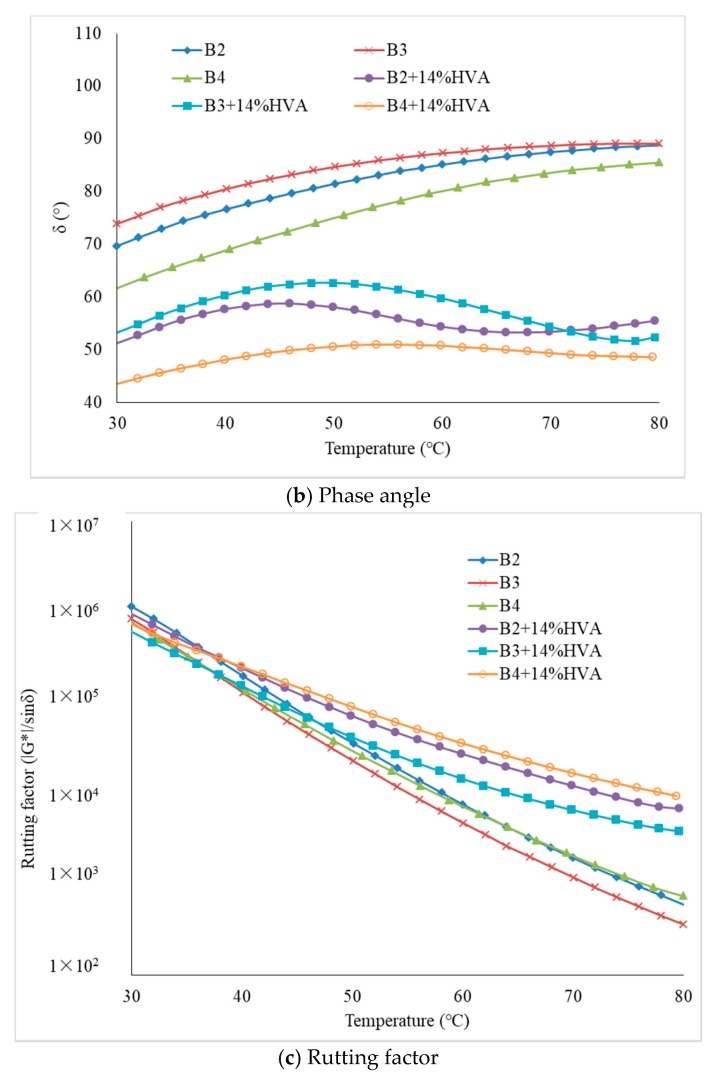
Temperature sweep test results.

DSR measurement is used for temperature sweep. The temperature sensitivity of different asphalt is analyzed by measuring complex shear modulus |G*|, phase angle δ and rutting factor |G*|/sinδ of asphalt. With a fixed loading frequency of 10 rad/s and a stress level of 0.1 kPa, temperature sweep test of the three kinds of matrix asphalt and corresponding high-viscosity modified asphalt with 14% HVA are carried out at test temperature from 30 °C to 80 °C. Parallel plate of 25 mm is used and the space between upper and lower parallel plates is fixed at 1 mm. For different kinds of asphalt, complex shear modulus |G*|, phase angle δ and rutting factor |G*|/sinδ in accordance with different temperature are shown in Figure 6.

As can be seen from Figure 6a, the dynamic shear modulus of all the asphalt samples have little difference from 30 °C to 45 °C. This is because of the low penetration and high consistency at low temperatures of matrix asphalt. When test temperature is higher than 45 °C, with the increase of temperature, the dynamic shear modulus of the three matrix asphalt decreases rapidly. For HVMA, with the increase of temperature, swelling effects between the HVA and light component in asphalt causes the increase of heavy component content in the asphalt, which in turn makes the asphalt viscous and hard macroscopically, meanwhile the flexibility is improved and higher value of shear modulus is obtained. The dynamic shear modulus of B4 + 14% HVA is the largest among the three types of high-viscosity modified asphalt.

In Figure 6b, it shows that phase angle of B4 is the smallest at the same test temperature among three matrix asphalt, which indicates that it has less viscous components and better resistance to permanent deformation. The phase angles of three matrix asphalt are greatly reduced after adding 14% of HVA, indicating that addition of HVA improves resistance ability to permanent deformation. Phase angles of three matrix asphalt gradually become larger as the test temperature increases, which shows more viscous properties. However, with the increase of temperature, phase angles of three high-viscosity asphalt firstly increase and then decrease, with small overall phase angle, showing greater elastic properties.

As can be seen from Figure 6c, with the increase of test temperature, rutting factor |G*|/sinδ of both matrix asphalt and high-viscosity asphalt added with HVA gradually decreases and the asphalt rutting factor and test temperature show an exponential decreasing trend. Compared with matrix asphalt, the three high-viscosity asphalt binders are less sensitive to temperature. As sweep temperature rises above 70 °C, the sequence of rutting factor value for different high-viscosity asphalt is: B4 + 14% HVA > B3 + 14% HVA > B2 + 14% HVA. 

### 3.3. Multiple Stress Creep Recovery Test

The multiple stress creep recovery (MSCR) test method is incorporated in AASHTO TP70 [15], which is a new-generation test method for evaluating elasticity resuming performance of modified asphalt. MSCR specimen is characterized by less test loading times and easy calculation of parameters [16,17]. A dynamic shear rheometer set with 25 mm parallel plate and 1 mm gap is used in MSCR test. Loading stress at the first stage is 0.1 kPa and that at the second stage is 3.2 kPa. Under controlled-stress mode, different stress levels are used for repeated loading-unloading tests of asphalt, with 10 cycles per stage. For each cycle, asphalt is loaded for 1 s and unloaded for 9 s. 

**Figure 7:**
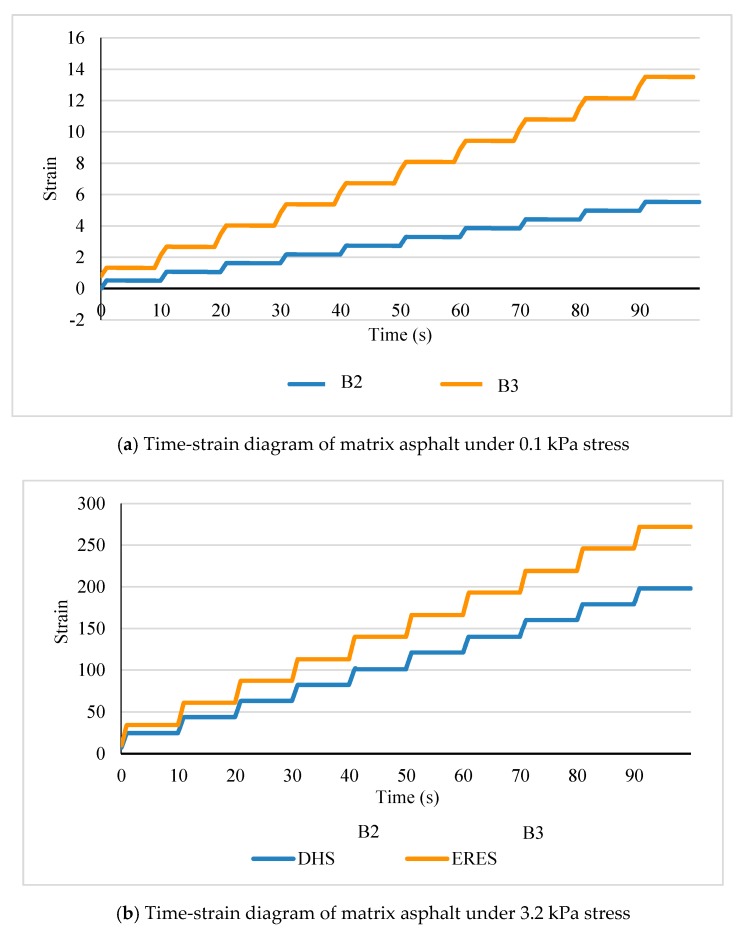
MSCR test results of matrix asphalt.

**Figure 8:**
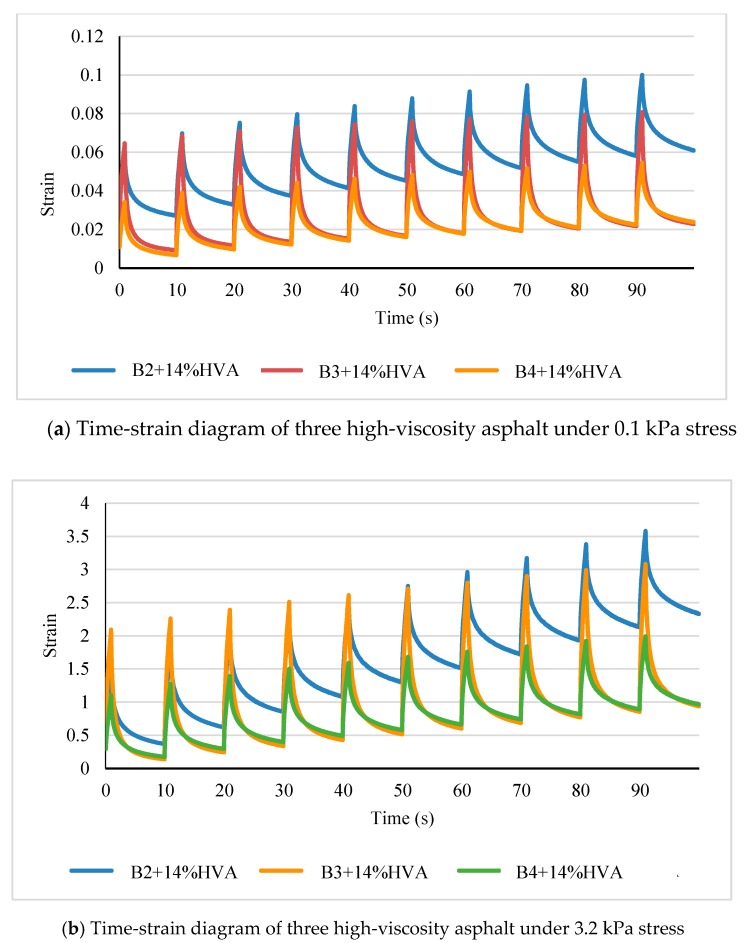
Test results of high-viscosity modified asphalt.

At the test temperature of 70 °C, the strains obtained at different loading times for the three matrix asphalt and high-viscosity modified asphalt after adding 14% HVA are shown in Figure 7 and Figure 8 respectively. 

As can be seen from Figure 7 and Figure 8, with increasing loadings cycles, strain of asphalt is enlarged and creep recovery decreases. Strain of the same kind of asphalt increases significantly under a larger stress level. When placed under load, matrix asphalt has greater strain than high-viscosity asphalt. Besides, after loading stage, matrix asphalt has weak recovery ability, without strong resistance to deformation. 

In AASHTO [15], deformation recovery capacity and high temperature rutting resistance of asphalt are evaluated by deformation recovery rate *R* and non-recoverable creep compliance *J_nr_*. Essentially, *J_nr_* represents the viscosity (non-recoverable) in creep compliance of the material. The two parameters are calculated as follows:

(3) $$R=(r_{p}-r_{nr})/(r_{p}-r_{0})$$

(4) $$J_{nr}=(r_{nr}-r_{0})/\tau$$

where $r_{p}$ represents peak strain in a loading cycle, $r_{nr}$ represents residual strain in a loading cycle, $r_{0}$ represents initial strain in a loading cycle and τ the shear stress.

*R* and *J_nr_* of the first 10 creep cycles are calculated according to Equations (3) and (4) respectively. Then the average creep recovery rate (*R*_0.1_) and average non-recoverable creep compliance (*J_nr_*_0.1_) at stress level of 0.1 kPa are obtained. In a similar way, average creep recovery rate (*R*_3.2_) and average non-recoverable creep compliance (*J_nr_*_3.2_) at stress level of 3.2 kPa can be obtained as well. The results of *R*_0.1_, *R*_3.2_, *J_nr_*_0.1_ and *J_nr_*_3.2_ of three matrix asphalt and high-viscosity asphalt with addition of 14% HVA are shown in Table 4. 

From Table 4, it is known that at the same temperature, there is a dramatic increase of recovery rate and a significant reduction of non-recoverable creep compliance after modification by 14% HVA. The creep recovery rate of B3 is negative at both 0.1 kPa and 3.2 kPa, which may be due to poor thermal stability of matrix asphalt and the test temperature of 70 °C exceeds its softening point. As stress level increases from 0.1 kPa to 3.2 kPa, recovery rate of all asphalt is reduced, which proves that actual pavement is more likely to produce rutting when subjected to higher pressure. At stress levels of 0.1 kPa and 3.2 kPa, the B3 + 14% shows highest deformation recovery capability among the three, which is mainly due to the greater content of aromatic and resin of the matrix asphalt. As to the non-recoverable creep compliance, the B4 + 14% modified asphalt presents a lowest value at both stress levels of 0.1 kPa and 3.2 kPa. This is because of a higher content of asphaltene in the matrix asphalt, which makes it less sensitive to a loading impact at high temperature. *J_nr_* indicates capability of asphalt to recover from deformation and its value has negative correlation with rutting resistance, that is, the higher is non-recoverable creep compliance, the poorer performance on deformation resistance of asphalt pavement. 

### 3.4. Accelerated Fatigue Test

**Figure 9:**
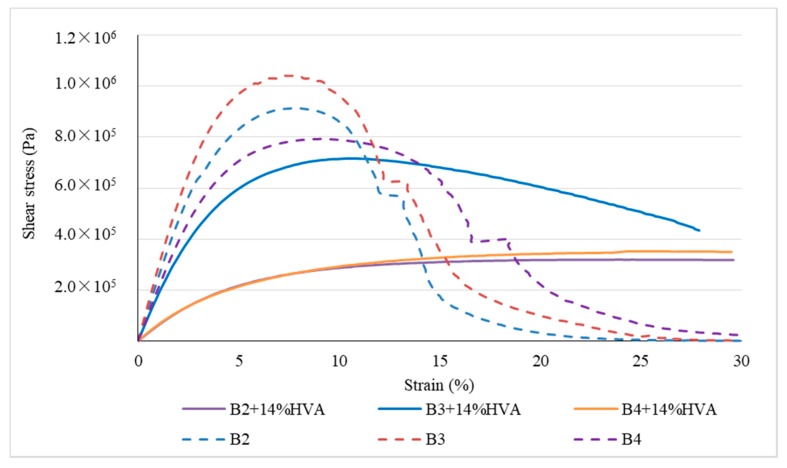
Stress-strain curves of three kinds of matrix asphalt and high-viscosity asphalt with 14% HVA.

The asphalt accelerated fatigue test [18] can quickly evaluate and predict asphalt fatigue performance to determine fatigue resistance of asphalt qualitatively. In this paper, DSR linear amplitude sweep (LAS) is used to test the shear stress with different shear strain value. The test method is as follows: firstly, short-term aging of asphalt is carried out and then measurement is carried out under the condition of 10 Hz, 20 °C (lower than the working temperature of asphalt in this area), strain sweep range 0.1%~30%, sweep rate LAS-5. The stress-strain curves of the three types of matrix asphalt and high-viscosity modified asphalt with 14% HVA are shown in Figure 9. 

From Figure 9, it can be seen that shear stress of matrix asphalt first appears a peak value with increase of strain, then it decreases continuously, which is mainly due to elastic and plastic deformation is generated in the process. The gradual decrease of shear stress after the peak value indicates that yield stress of asphalt occurs under corresponding strain, which is called yield strain. In general, the higher the yield strain, the better the elastic properties are. 

The high stress levels are observed when the strain is between 5% and 10% and the decline is relatively rapid. After addition of 14% HVA, B3 asphalt can maintain high stress for a larger range of strain in comparison with the matrix asphalt B2. B2 + 14% HVA and B4 + 14% HVA asphalt do not show a decreasing trend of stress within 30% of the strain and does not reach the fatigue failure point within 30% strain range, which indicate a better fatigue resistance performances after modified by HVA.

## 4. Study on Viscosity-Temperature Characteristics of Low-Grade High-Viscosity Modified Asphalt

This section mainly aims at appropriate temperature range for the construction of porous asphalt with different high-viscosity modified asphalt. For porous asphalt mixture prepared by high-viscosity modified asphalt, the viscosity range of ordinary asphalt cannot be used for determining the construction temperature range. So a new method is needed to determine mixing and compaction temperatures of the mixtures.

The relationship between viscosity and temperature of high-viscosity modified asphalt can be expressed by the Saal formula:

(5) $$\mathrm{lglg}\eta \times 10^{3}=n-m\mathrm{lg}\left(T+271.13\right)$$

where,

*T*—Temperature, °C:
*η*—Viscosity, Pa·s:
*n*—Regression constant, the higher the value, the greater the viscosity at the same temperature;
*m*—Regression constant, indicating asphalt temperature sensitivity. The larger the absolute value, the worse the temperature sensitivity.

**Figure 10:**
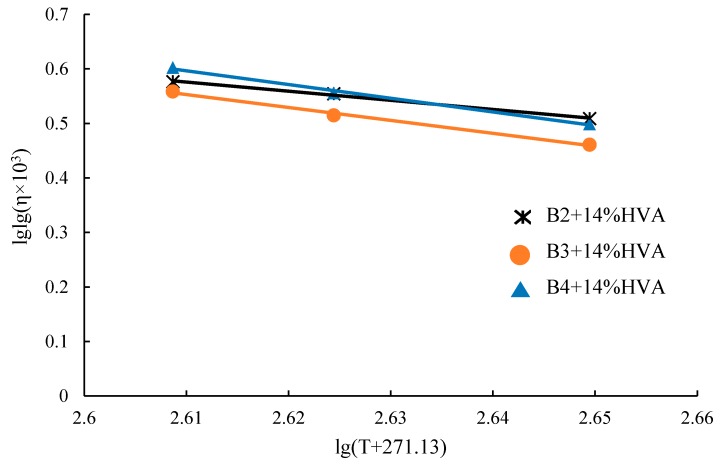
Viscosity-temperature curve of three asphalt with 14% HVA on double logarithmic coordinates.

Brookfield viscosity of three types of HVMA (Asphalt: HVA= 86:14) at 135 °C, 150 °C, 175 °C are measured by Brookfield viscometer and the curve of regression by Saal formula is shown in Figure 10. 

According to the relation given by Saal formula and based on above data, the viscosity-temperature curves of three different asphalt with 14% HVA are as follows:

B2 + 14%HVA: y = −2.3404x + 6.5527 (R^2^ = 0.9942) B3 + 14%HVA: y = −3.4245x + 9.3507 (R^2^ = 0.9968) B4 + 14%HVA: y = −3.4914x + 9.5844 (R^2^ = 0.9964) (6)

From existing studies, the viscosity range for mixing and compaction temperatures of modified asphalt are considered [19]:

Mixing temperature: η=0.275±0.03Pa·s

Compaction temperature: η=0.550±0.04Pa·s

Based on the viscosity range above and the viscosity-temperature curve, the mixing and compaction temperatures of HVMA with 14% HVA are obtained and shown in Table 5. From the table, it can be seen that HVMA has a relatively high-viscosity at low temperature in use such as 60~70 °C but temperatures required for mixing and compaction are not too high. Besides, porous asphalt mixture generally has coarse aggregates content, it is favorable for the high-viscosity additive to be grinded in the mixing. For B3 + 14% HVA, considering the heating temperature and the workability of the mixing, it is also suggested the mixing temperature to be 160 °C. Then, from the study, the mixing temperature for HVMA from low grade asphalt is considered to be 160 °C and the compaction temperature is between 130 °C and 145 °C, depending on the matrix asphalt type.

## 5. Conclusions and Recommendations

Performances of high-viscosity modified asphalt prepared by typical low grade asphalt in Africa were studied in this research. The conclusions drawn from the research are as follows:

(1) According to temperature characteristics of rainy environments in Africa, the lower limit of dynamic viscosity at 60 °C is considered to be 270,000 Pa·s for satisfying the rutting resistance requirement.
(2) The high-viscosity modification process can greatly improve PG high temperature grade and high rutting resistance of the low grade asphalt, as well as reducing the sensitivity of asphalt to loading frequency.
(3) For matrix asphalt with higher asphaltene content, a larger dynamic viscosity at 60 °C is achieved after high-viscosity modification and it also shows relatively good resistance to deformation caused by high temperature and shearing. For a matrix with lower asphaltene content but higher aromatic and resin, the most outstanding elastic recovery capability can be obtained after high-viscosity modification.
(4) For application in porous asphalt in Africa, the suitable mixing temperature is considered to be 160 °C, while the compaction temperature is suggested to be from 130 °C to 145 °C for various types of high-viscosity modified asphalt binders.

The research can help guide the design of porous asphalt mixture based on low-grade and high-viscosity modified asphalt and provide a basis for implementation on the trial section of asphalt pavement in Africa. Studies on mixtures by using high-viscosity modified asphalt and observations of trial section will be further carried out in future work.

## Figures and Tables

**Table 1:** Test results on matrix asphalt.

Items	Unit	Technical Requirement	Test Value			
			B1	B2	B3	B4
Penetration (25 °C, 100 g, 5 s)	0.1 mm	30–45	44	37	40	41.1
Softening point values (TR&B)	°C	52–60	54	53.5	50.8	55.4
Flashpoint	°C	≥240	322	316	308	346
Solubility	%	≥99	99.79	99.92	99.69	99.83
Dynamic viscosity (60 °C)	Pa·s	≥260	485	394	265	566
Residue mass change after TFOT	%	≤0.5				
Residual penetration (25 °C)	0.1 mm	-	31	25.9	32.6	27.5
Penetration ratio	%	≥53	70.5	70	81.5	67

**Table 2:** Test results of four-component for matrix asphalt.

Asphalt Type	Asphaltene (%)	Saturate (%)	Aromatic (%)	Resin (%)
B1	20.74	13.93	39.20	23.50
B2	16.10	9.80	47.32	25.43
B3	11.28	8.26	51.35	28.84
B4	17.05	14.16	40.03	27.41

**Table 3:** Test results of high temperature performance grade.

Test Temperature (°C)	B2	B2 + 14%HVA	B3	B3 + 14%HVA	B4	B4 + 14%HVA
Original asphalt(|G*|/sin(δ) (|KPa|))						
58	8.51	-	5.87	21.8	10.9	-
64	3.6	-	2.47	11.5	4.71	-
70	1.63	-	1.13	7.26	2.14	-
76	0.779	-	0.55	5.26	1.04	9.93
82	-	4.84	-	4.14	-	6.9
88	-	3.15	-	3.33	-	5.04
After short-term aging(|G*|/sin(δ) (|KPa|))						
58	13.4	-	10.1	-	22.8	-
64	5.59	-	4.04	-	9.81	-
70	2.48	-	1.76	-	4.39	-
76	1.16	-	-	-	2.06	-
82	-	4	-	2.74	-	7.97
88	-	2.31	-	1.68	-	4.61

**Table 4:** Results of creep recovery rate and non-recoverable creep compliance.

Asphalt Type	Parameters			
	*R*_0.1_ (%)	*J_nr_*_0.1_ (kPa^−1^)	*R*_3.2_ (%)	*J_nr_*_3.2_ (kPa^−1^)
B2	0.0265	27.5500	0.0068	31.9725
B2 + 14%HVA	0.8926	0.7057	0.8426	0.6644
B3	−0.0199	40.1736	−0.0201	43.9400
B3 + 14%HVA	0.9570	0.3833	0.9427	0.6235
B4	0.0030	37.4957	−0.0422	47.7564
B4 + 14%HVA	0.9091	0.1650	0.8836	0.3931

**Table 5:** Mixing and compaction temperature for different HVMA.

Asphalt Type	Mixing Temperature (°C)	Compaction Temperature (°C)
B2 + 14%HVA	160	139
B3 + 14%HVA	143	130
B4 + 14%HVA	160	145
